# Overexpression of *RNF146* in Non-Small Cell Lung Cancer Enhances Proliferation and Invasion of Tumors through the Wnt/β-catenin Signaling Pathway

**DOI:** 10.1371/journal.pone.0085377

**Published:** 2014-01-14

**Authors:** Ying Gao, Chengyang Song, Linping Hui, Chun-yu Li, Junying Wang, Ye Tian, Xu Han, Yong Chen, Da-Li Tian, Xueshan Qiu, Enhua Wang

**Affiliations:** 1 Department of Pathology, The First Affiliated Hospital and College of Basic Medical Sciences of China Medical University, Shenyang, Liaoning, China; 2 Department of Pathology, The Fourth Affiliated Hospital of China Medical University, Shenyang, Liaoning, China; 3 Department of Thoracic Surgery,The Fourth Affiliated Hospital of China Medical University, Shenyang, Liaoning, China; 4 Laboratory Center, The Fourth Affiliated Hospital of China Medical University, Shenyang, Liaoning, China; Cincinnati Children’s Hospital Medical Center, United States of America

## Abstract

Studies have suggested a possible correlation between the newly identified E3 ubiquitin ligase ring finger protein 146 (RNF146) and tumor development. However, until now, studies on RNF146 have been restricted to poly(ADP-ribosyl)ation and ubiquitin ligation, whereas the role of RNF146 in tumor biology has rarely been reported. In the present study, the role of RNF146 in non-small cell lung cancer (NSCLC) was investigated. The results showed that the expression of RNF146 was increased in clinical lung cancer samples and cell lines. RNF146 expression correlated with tumor size, differentiation level, lymphatic metastasis, pTNM staging, and prognosis of patients in stage I. RNF146 expression was negatively correlated with Axin expression but positively correlated with the nuclear expression of β-catenin in NSCLC tissues. RNF146 downregulated the expression of Axin in lung cancer cell lines and induced the expression and nuclear distribution of β-catenin. Overexpression of RNF146 in NSCLC cell lines increased the levels of cyclinD1, cyclinE, and CDK4, promoted cell cycle G_0_/G_1_-S transitions, and regulated cell proliferation. Overexpression of RNF146 led to upregulated levels of matrix metalloproteinases 2 and 7 and enhanced lung cancer cell invasiveness, events that were mediated by the classical Wnt/β-catenin signaling pathway. In summary, the data in the present study indicate that RNF146 regulated the development and progression of NSCLC by enhancing cell growth, invasion, and survival, suggesting that RNF146 may be a potential treatment target in NSCLC.

## Introduction

E3 ubiquitin ligases play important roles in regulating cell functions including proliferation, cell cycle arrest, and apoptosis. They may also have additional functions that depend on the identity of their substrates. For example, if an E3 ubiquitin ligase targets an oncogene for degradation, it may be considered a tumor suppressor. Similarly, if an E3 uniquitin ligase degrades a tumor suppressor protein, it may be considered an oncogene. Many proteins containing RING-finger domains possess ubiquitin ligase activity, some of which participate in tumorigenesis and tumor metastasis. The newly identified E3 ubiquitin ligase RING finger protein 146 (RNF146) interacts with poly(ADP-ribose) (PAR) through a PAR-binding motif in the Trp-Trp-Glu (WWE) domain. The *RNF146* gene is located on human chromosome 6q22, 33 [1]. RNF146 has neuroprotective activity due to its inhibition of Parthanatos via binding with PAR [2]. RNF146 may facilitate DNA repair against cell death induced by DNA-damaging agents or γ-irradiation [3]. In response to cellular damage, RNF146 translocates to the nucleus and enhances the ubiquitination and degradation of various nucleoproteins that participate in DNA damage repair. In addition, as a poly(ADP-ribosyl)ation (PARsylation)-directed E3 ubiquitin ligase, RNF146 regulates the Tankyrase-dependent degradation of Axin and positively regulates the Wnt signaling pathway [1].

The Wnt signaling pathway is highly active in lung cancer cells, leading to metastasis and proliferation of these cells [4]. Wnt signaling can be aberrantly activated by various mechanisms, and a main function is to inhibit the proteolysis of β-catenin, which is controlled by phosphorylation [5]. Free β-catenin can enter the nucleus and activate the target genes of Wnt. Steady-state levels of Axin are very important, as this scaffolding protein initiates formation of the β-catenin degradation complex. Researchers have demonstrated that the transfer of PAR to residues of Axin catalyzed by Tankyrase leads to the PARsylation of Axin [1], [6]. RNF146 participates in the degradation of PARsylated Axin through its PAR-binding motif. This interaction leads to destruction of the β-catenin degradation complex, aggregation of intracellular β-catenin, and increased signaling through the Wnt pathway [1].

Despite many studies on RNF146, its exact role in tumorigenesis remains unclear. In the present study, the roles of RNF146 in lung cancer were investigated.

## Materials and Methods

### NSCLC Tissue Samples

Primary NSCLC samples and control tissues were collected from 133 patients. Normal lung samples were taken from lung tissue more than 5 cm from the cancer resection site. Procedures took place at the Fourth Affiliated Hospital of China Medical University. The patients did not receive any radiation or chemotherapy before the operation. NSCLC staging was based on the TNM Classification of Malignant Tumors, Seventh Edition [7]. The survival time was calculated from the operation day to death via the evaluation of recurrence and metastasis or until the last follow-up date. Fresh specimens were frozen in liquid nitrogen and stored at −80°C. For experiments involving human tissues, approval was obtained from the institutional review committee of China Medical University. Written informed consent was provided according to the Declaration of Helsinki.

### Antibodies and Reagents

The rabbit anti-human RNF146 polyclonal antibody was purchased from Abcam (Cambridge Science Park). Anti-Axin and anti-β-catenin antibodies were purchased from BD Transduction Laboratories (San Jose, CA, USA). Anti-CyclinA, anti-CyclinB, anti-CyclinD1, and anti-pRB antibodies were from Cell Signaling Technology, Inc. (Boston, MA, USA). Anti-CDK4, Anti-CDK6, Anti-TIMP-1, Anti-CyclinE, siAxin, siβ-catenin, and siTCF4 were from Santa Cruz Biotechnology (Santa Cruz, CA, USA). Antibodies against RhoA, RhoB, RhoC, and Rock1 were from Proteintech (Chicago, IL 60612, USA). Antibodies against CDK2, P21, matrix metalloproteinase 2 (MMP2), MMP7, and MMP9 were from NeoMarkers (Palo Alto, CA, USA). Anti-P27 and anti-P53 were from Thermo Fisher Scientific (Fremont, CA, USA). The recombinant overexpression plasmids pEX4-hRNF146 and pGPHI-shhRNF146 and control empty plasmids were constructed by GenePharma (Shanghai, China). The immunohistochemical secondary antibody kit was purchased from Dako (Copenhagen, Denmark).

### Cell Lines and Cell Culture

The normal bronchial epithelial cell line HBE, the human lung adenocarcinoma cell lines A549 and H1299 were purchased from the American Type Culture Collection (Manassas, VA, USA). The human NSCLC cell lines LK2, LH7, LTE, and SPC were purchased from the Shanghai Cell Bank of the Chinese Academy of Science. Cell lines were cultured in Kaighn’s Modification of Ham’s F-12 Medium, Roswell Park Memorial Institute (RPMI)-1640 or Dulbecco’s modified Eagle’s-F12 medium with 10% fetal bovine serum (Invitrogen, Carlsbad, CA, USA) containing 100 units/ml penicillin and 100 units/ml streptomycin (Sigma, St. Louis, MO, USA) at 37°C with 5% CO_2_.

### Immunohistochemical Staining

Paraffin sections with a thickness of 4 µm were prepared and immunohistochemically stained with Envision as described previously [8]–[9]. Paraffin specimens were incubated with anti-RNF146 (1∶100), anti-Axin (1∶200), and anti-β-catenin (1∶200) antibodies at 4°C overnight. PBS-incubated samples were used as negative controls.

Two pathology physicians assessed the staining results on a semiquantitative scale. Five high magnification fields were randomly selected from each section, and 100 tumor cells in each field were counted to evaluate the intensity and range of RNF146 staining. A negative result was given a score of 0 (no staining), faint yellow staining was recorded as a 1, yellow was scored as a 2, and deep brown staining was recorded as a 3. Percentage scores were assigned as follows: <5%, 0; 5–25%, 1; 26–50%, 2; 51–75%, 3; ≥75%, 4. Two independent scores were multiplied together to obtain the patient’s coloring coefficient, which was categorized as “low expression” for coloring coefficient <4 and “high expression” for coloring coefficient ≥4. Axin and β-catenin were scored as previously described [8]–[9].

### Cell Transfection

Cells were plated on 24-well plates at approximately 50% confluence and transfected with plasmid or siRNA 24 hours later. Transfection of lung cancer cell lines A549 and LTE was performed using Lipofectamine 2000 (Invitrogen) according to the manufacturer’s directions. For co-transfection experiments, 1 to 1.5 µg of co-transfected or empty vector and 45 pmol siRNA were added to the transfection mixture. After 5 h at 37°C and 5% CO_2_, complete medium was added to the transfection mixture. Transfection efficiency was determined by Western blot.

### RT-PCR Assay

Total RNA was extracted using TRIzol (Invitrogen) according to the directions in the TaKaRa RNA PCR Kit(TaKaRa, Dalian, China). GAPDH expression served as the internal control. The primer sequences were: RNF146: forward: 5′-GGACGTCGCAGGAAGATTAAG-3′ and reverse: 5′-CAATGGAGGTGTCTGGTGCT-3′; Axin: forward: 5′-GACTTGCTGGACTTCTGGTT-3′ and reverse: 5′-TGTACTTTCGGTAGATGGCT-3′; β-catenin: forward: 5′-CTAAACAGGAAGGGATGGAAG-3′ and reverse: 5′-ACAGATGGCAGGCTCAGTGAT-3′; GAPDH: forward: 5′-GAAGGTCGGAGTCAACGGAT-3′ and reverse: 5′-CCTGGAAGATGGTGATGGG-3′. The PCR products for RNF146 (376 bp), Axin (109 bp), β-catenin (221 bp), and GAPDH (153 bp) were amplified with 30 PCR cycles.

### Western Blot

Cells were lysed in buffer containing 150 mM NaCl, 1% NP-40, 0.1% SDS, 2 µg/ml aprotinin, and 1 mM PMSF. Total protein was harvested, and 60 µg were separated by SDS-PAGE and transferred to polyvinylidene fluoride (PVDF) membranes (Millipore, Billerica, MA, USA). Proteins on PVDF membranes were blocked with 5% bovine serum albumin (BSA) in phosphate buffered saline with tween (PBST), incubated with primary antibody at 4°C overnight, and then incubated with secondary antibodies at room temperature for 2 hours. Membranes were developed with enhanced chemical liquid (ECL) (Thermo Fisher Scientific), and the results were recorded using a bioimaging system (UVP Inc., Upland, CA, USA). Protein expression levels were evaluated using β-actin as a loading control.

### Immunofluorescence Staining

Cells grown on glass cover slips were fixed with 4% paraformaldehyde and treated with 0.2% Triton X-100. After washing with PBS, cells were blocked with nonimmunized animal serum at 37°C for 30 minutes, and then incubated with primary antibodies anti-RNF146 (1∶100), anti-Axin (1∶100), and anti-β-catenin (1∶100) at 4°C overnight. Negative and positive controls were included in all experiments. Cells were incubated with FITC or TRITC-labeled secondary antibodies (1∶100, Chemicon, USA), at 37°C for 30 minutes. After washing with PBS, cells were incubated with 0.1% 4′, 6-diamidino-2-phenylindole (DAPI) (Invitrogen) at 37°C for 10 minutes. Cells were washed with PBS and observed under a fluorescence microscope.

### MTT Assay

Transfected cells and control cells were plated on 96-well plates at 10^3^ cells per well and cultured for 24 hours. Cell proliferation was detected with the cell counting Kit-8 solution (Dojindo, Gaithersburg, MD) according to the manufacturer’s instructions. The 3-(4,5-Dimethylthiazol-2-yl)-2,5-diphenyltetrazolium bromide (MTT) assay was repeated on 4 consecutive days. Optical density values were measured using a microplate reader (Spectra Thermo, Männedorf, Switzerland) at an absorbance of 550 nm.

### Wound-healing Assay

Each well of a 6-well plate was seeded with 5×10^5^ cells, cultured for 24 hours, and scratched with a spearhead. Cells were washed with PBS and cultured in serum-free medium at 37°C and 5% CO_2_. Samples were harvested and photographs were taken at 0, 6, 12 and 24 hours. Each experiment was repeated at least three times and average values were calculated.

### Transwell

Cell suspensions of 100 µl containing 2.5×10^6^ cells/ml were added to the upper chamber of a transwell insert, and 600 µl medium containing 10% fetal bovine serum was placed in the lower chamber. A polycarbonate microporous membrane with a diameter of 8 µm separated the upper and the lower chambers. Cells were cultured at 37°C and 5% CO_2_ for 6 hours. After washing with PBS, cells were fixed with methanol at room temperature for 15 minutes. Finally, the treated cells were stained with hematoxylin, photographs were taken, and stained cells were counted. To measure the invasive capacity of cells, precooled medium without serum and Matrigel (BD Biosciences, USA) were mixed at a volume ratio of 1∶7 and were added into the upper chamber. The volume of the mixture liquid was 100 µl. The cells were treated for 3 hours at room temperature, and the results were obtained after the cells were cultured for 24 hours.

### Cell Cycle Assay

Cells were seeded at 5×10^6^ cells per 6 cm culture dish, cultured for 12 hours, and transfected. Cells were synchronized by serum starvation for 20 hours. Cells were maintained in medium containing 10% serum for 24 hours. Cells were harvested and fixed in cold 70% ethanol. After washing with PBS, cells were incubated with 0.1 mg/ml RNaseA (Roche Indianapolis, IN) at 37°C for 30 minutes and treated with 5 mg/ml propidium iodide for 30 minutes. Finally, the treated cells were analyzed by flow cytometry.

### Statistical Analysis

Databases were set up using Excel. Data analysis was conducted using SPSS17.0. Relationships between the expression of RNF146, Axin, β-catenin, and clinical and pathological factors were evaluated by the *χ^2^* test and Fisher test. Kaplan-Meier survival curves and Log-rank were used to detect and evaluate differences. Other data were compared by *t*-test. Data from three independent experiments were shown as 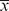 *±s*. A *P*<0.05 was considered statistically significant.

## Results

### RNF146 Overexpression in NSCLC

**Figure 1:**
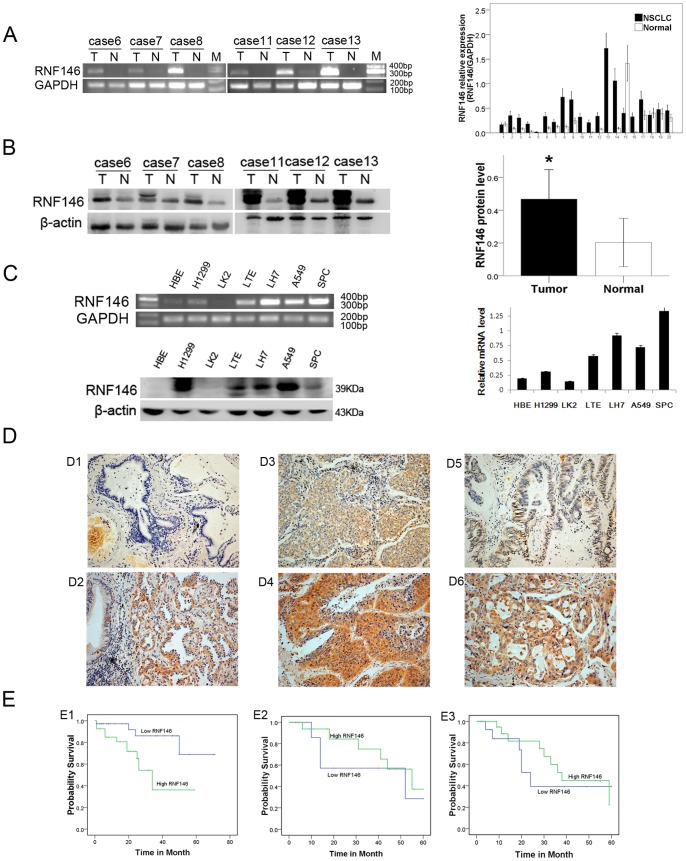
RNF146 overexpression in NSCLC. Detection of mRNA and protein expression of RNF146 in 20 NSCLC and control samples by RT-PCR (A) and Western blot (B). T: tumor samples; N: matched normal tissues; M: Marker. (C) mRNA and protein expression of RNF146 in the HBE cell line and the human NSCLC cell lines LK2, LH7 (squamous cancer), H1299, LTE, A549, and SPC (adenocarcinoma). GAPDH and β-actin served as internal controls. (D) Expression and distribution of RNF146 in NSCLC by immunohistochemistry. D1, RNF146 was not expressed in the normal bronchial and alveolar epithelium. D2, RNF146 was expressed in the cytoplasm of lung cancer cells, and weak RNF146 expression was detected in the adjacent bronchial epithelium. D3 and D5 showed weak RNF146 staining in the well-differentiated squamous cell carcinoma and adenocarcinoma. D4 and D6 showed strong RNF146 staining in the poorly differentiated squamous cell carcinoma and adenocarcinoma. (E) Relationship between RNF146 expression and the prognosis of NSCLC patients. E1 showed correlation between RNF146 expression and prognosis of stage I patients (*P*<0.05). E2 and E3, no correlation between RNF146 expression and prognosis of stage II and stage III patients (*P>*0.05).

To investigate the expression of RNF146 in NSCLC, 20 paired NSCLC and non-cancerous tissue samples were collected, and RT-PCR and Western blot were used to analyze the RNF146 mRNA and protein expression levels, respectively. Expression of RNF146 mRNA in the NSCLC samples was higher than that of the non-cancerous lung tissues (*P = *0.030) (Figure 1A). The results of the Western blot and immunohistochemical staining confirmed that RNF146 protein expression was increased in NSCLC (*P = *0.014, *P = *0.000) (Figure 1B), consistent with the RT-PCR results. The expression of RNF146 was also analyzed in the HBE and NSCLC cell lines. Different expression levels of RNF146 were observed among the NSCLC cell lines (Figure 1C). The expression of RNF146 mRNA and protein in both the HBE and the LK2 (squamous) cell lines were low. The expression of RNF146 protein in the LTE (adenocarcinoma) and LK2 (squamous) cell lines were moderate, and the expression of RNF146 protein in the A549 and H1299 (adenocarcinoma) cell lines were high. The data indicated that expression of RNF146 was increased in the clinical NSCLC samples and lung cancer cell lines.

To explore the role of RNF146 in NSCLC further, the relationships between protein expression and clinical pathological characteristics were analyzed. There were correlations between the expression of RNF146 and tumor size (*P* = 0.044), histological type (*P = *0.005), poor differentiation (*P* = 0.002), lymphatic metastasis (*P* = 0.009), and pTNM stage (*P* = 0.015). However, RNF146 expression was not correlated with the age or gender of NSCLC patients (Table 1, Figure 1D). Furthermore, Kaplan-Meier survival analysis demonstrated that overexpression of RNF146 was correlated with the survival of lung cancer patients in stage I (*P* = 0.016). There was no correlation between RNF146 expression and survival time in all patients (*P = *0.616) or those patients with stage II (*P* = 0.437), or stage III NSCLC (*P = *0.520) (Figure 1E).

**Table 1:** Correlation between RNF146 expression and clinicopathological factors of NSCLC.

Clinical pathological characteristics	Number of patients	RNF146 weak or negative	RNF146 High expression	*P*
**Age**				0.156
<60	53	20 (37.7%)	33 (62.3%)	
≥60	80	41 (51.3%)	39 (48.7%)	
**Gender**				0.110
Male	99	41 (41.4%)	58 (58.6%)	
Female	34	20 (58.8%)	14 (41.2%)	
**Histology type**				0.005*
Squamous cancer	62	20 (32.3%)	42 (67.7%)	
Adenocarcinoma	71	41 (57.7%)	30 (42.3%)	
**Differentiation**				0.002*
Well	39	26 (66.7%)	13 (33.3%)	
Moderate-Poor	94	35 (37.2%)	59 (62.8%)	
**Tumor status**				0.044*
T1	46	27 (58.7%)	19 (41.3%)	
T2T3T4	87	34 (39.1%)	53 (60.9%)	
**Nodal status**				0.009*
N0	73	41 (56.2%)	32 (43.8%)	
N1N2N3	60	20 (33.3%)	40 (66.7%)	
**TNM stage**				
I	69	39 (56.5%)	30 (43.5%)	0.015*
II+III	64	22 (34.4%)	42 (65.6%)	

*P*<0.05.

### Expression of RNF146, Axin, and Nuclear Distribution of β-catenin

**Figure 2:**
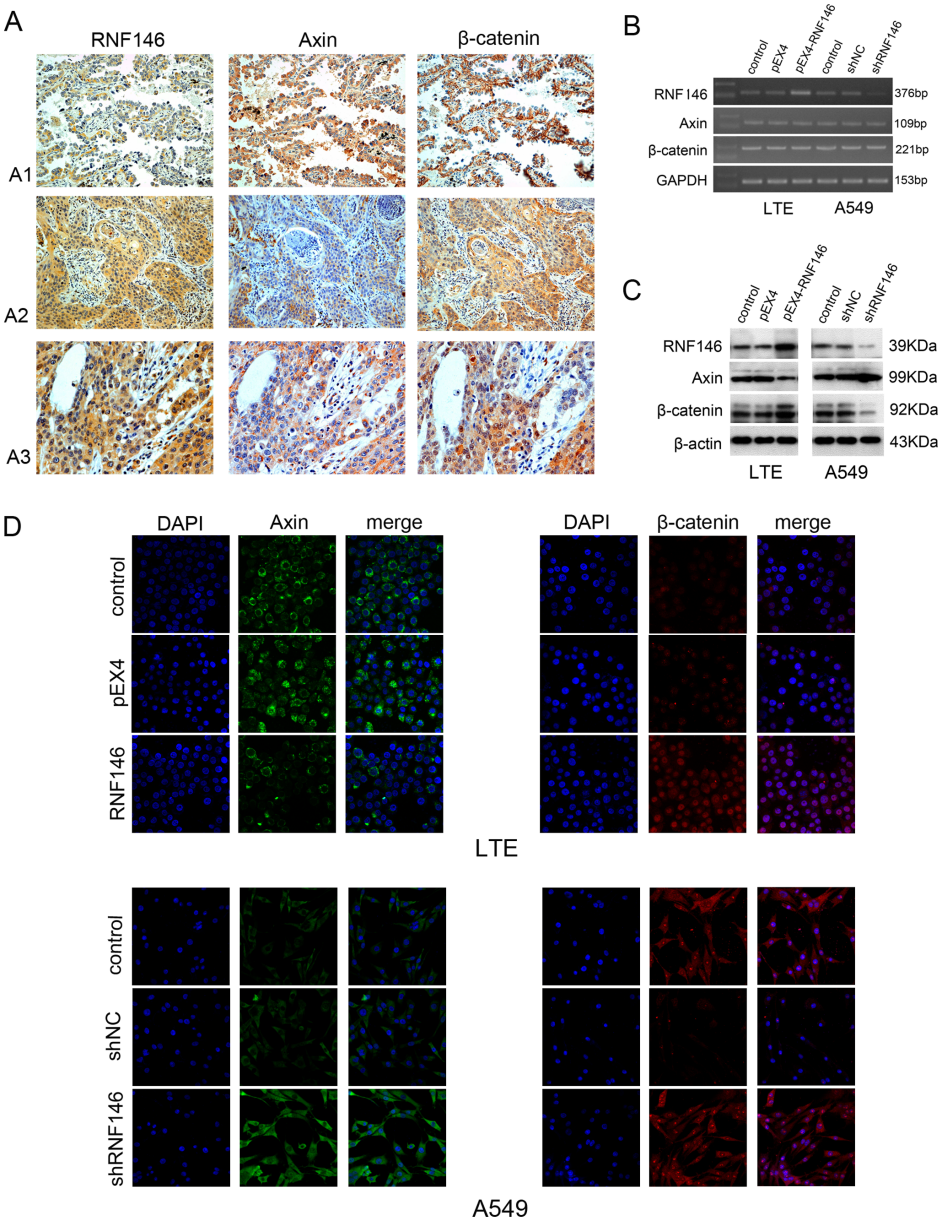
Expression of RNF146 and Axin and nuclear distribution of β-catenin. (A) Expression and distribution of RNF146, Axin, and β-catenin in NSCLC by immunohistochemistry (x 200). Strong Axin expression and membrane associated β-catenin correlated with low expression of RNF146 in adenocarcinoma (A1). Low Axin expression and cytoplasmic and nuclear-expression of β-catenin was found in squamous cancers with high expression of RNF146 (A2 and A3). Expression and distribution of RNF146, Axin and β-catenin detected by (B) RT-PCR, (C) Western blot, and (D) immunofluorescence. Cells transfected with empty vectors served as controls.

As an E3 ubiquitin ligase, RNF146 regulates the Tankyrase-dependent degradation of Axin and positively regulates the Wnt signaling pathway [1]. To investigate the relationship between RNF146, Axin, and β-catenin, the expressions of Axin and β-catenin in 133 NSCLC samples were detected by immunohistochemical methods and correlations of the proteins were calculated. RNF146 was negatively correlated with Axin expression (*P = *0.003), but it was not correlated with the decreased expression of β-catenin in membranes or the increased expression of β-catenin in the cytoplasm (*P = *0.524). High RNF146 expression was positively correlated with nuclear β-catenin staining (*P = *0.047) (Table 2, Figure 2A).

**Table 2:** Correlation of RNF146 and Axin and β-catenin expression in NSCLC tumors.

RNF146 expression	Axin expression (%)			β-catenin Nuclear expression(%)		
	Reduced	Preserved	*P*	Positive	Negative	*P*
**High**	49(68.1)	23(31.9)	0.003	19(26.4)	53(73.6)	0.047
**Low**	26(42.6)	35(57.4)		7(11.5)	54(88.5)	

LTE cell lines were transiently transfected with the pEX4-RNF146 overexpression plasmid or the control pEX4 empty plasmid. Experiments confirmed that RNF146 was upregulated. A549 and H1299 cell lines were transiently transfected with RNF146-shRNA1, RNF146-shRNA2 or control shNC, and experiments confirmed that RNF146 expression was reduced (Figure 2C and Figure S1A). Furthermore, expression levels of Axin and β-catenin were analyzed by RT-PCR, Western blot, and immunofluorescence. Overexpression or knock-down of RNF146 did not affect the mRNA expression levels of Axin and β-catenin (Figure. 2B). However, Western blot results indicated that the overexpression of RNF146 in LTE lung cancer cells inhibited the protein expression of Axin and increased the expression of β-catenin (Figure. 2C). Immunofluorescence further confirmed that overexpression of RNF146 decreased the cytoplasmic expression of Axin and increased the expression of β-catenin in the cytoplasm and nucleus. In contrast, reduced expression of RNF146 led to increased expression of Axin in the cytoplasm and decreased expression of β-catenin in the cytoplasm and nucleus (Figure. 2D).

### Expression of RNF146 and the Proliferation of Lung Cancer Cells

**Figure 3:**
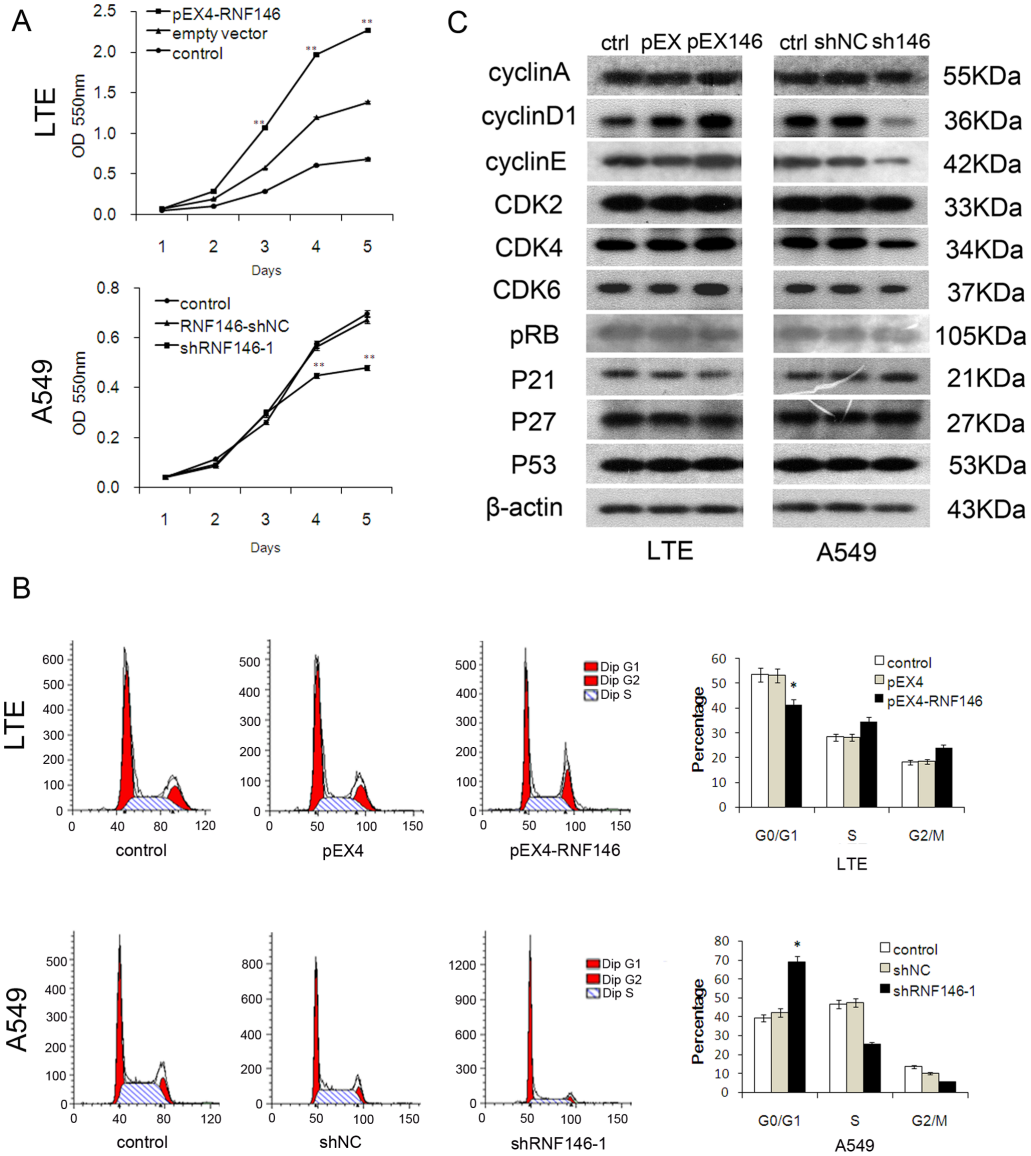
RNF146 regulated cell proliferation of lung cancer cells. (A) MTT cell proliferation assay with overexpression of RNF146 and shRNA-mediated knockdown of RNF146. **P<0.05. (B) Effects of RNF146 on cell cycle analyzed by flow cytometry. (C) Detecting cell cycle protein expression by Western blot.

To explore the roles of RNF146 in cell proliferation, LTE and A549 cells were transfected with plasmids, as described above, and cell proliferation was detected by the MTT assay. Compared with the control group and the group transfected with the empty plasmid, overexpression of RNF146 significantly enhanced cell proliferation in LTE cells, whereas knockdown of RNF146 inhibited cell proliferation in A549 and H1299 cells (Figure 3A and Figure S1B). The cell cycle was analyzed by flow cytometry, and the results demonstrated that overexpression of RNF146 decreased the percentage of G_0_/G_1_ phase LTE cells and increased the percentage of S phase cells. In contrast, knockdown of RNF146 in A549 and H1299 cells increased the percentage of cells in the G_0_/G_1_ phase and decreased the percentage of cells in S stage (Figure 3B and Figure S1C).

Western blot results also revealed correlations between the levels of RNF146 in lung cancer cells and the expression of cell cycle-related regulatory proteins including cyclinD1, cyclinE and CDK4 (Figure 3C). The data indicated that RNF146 regulated the expression of cyclinD1 and cyclinE and regulated cell cycle progression by inducing the G_0_/G_1_-S transition.

### RNF146 Expression and Invasion of Lung Cancer Cells

**Figure 4:**
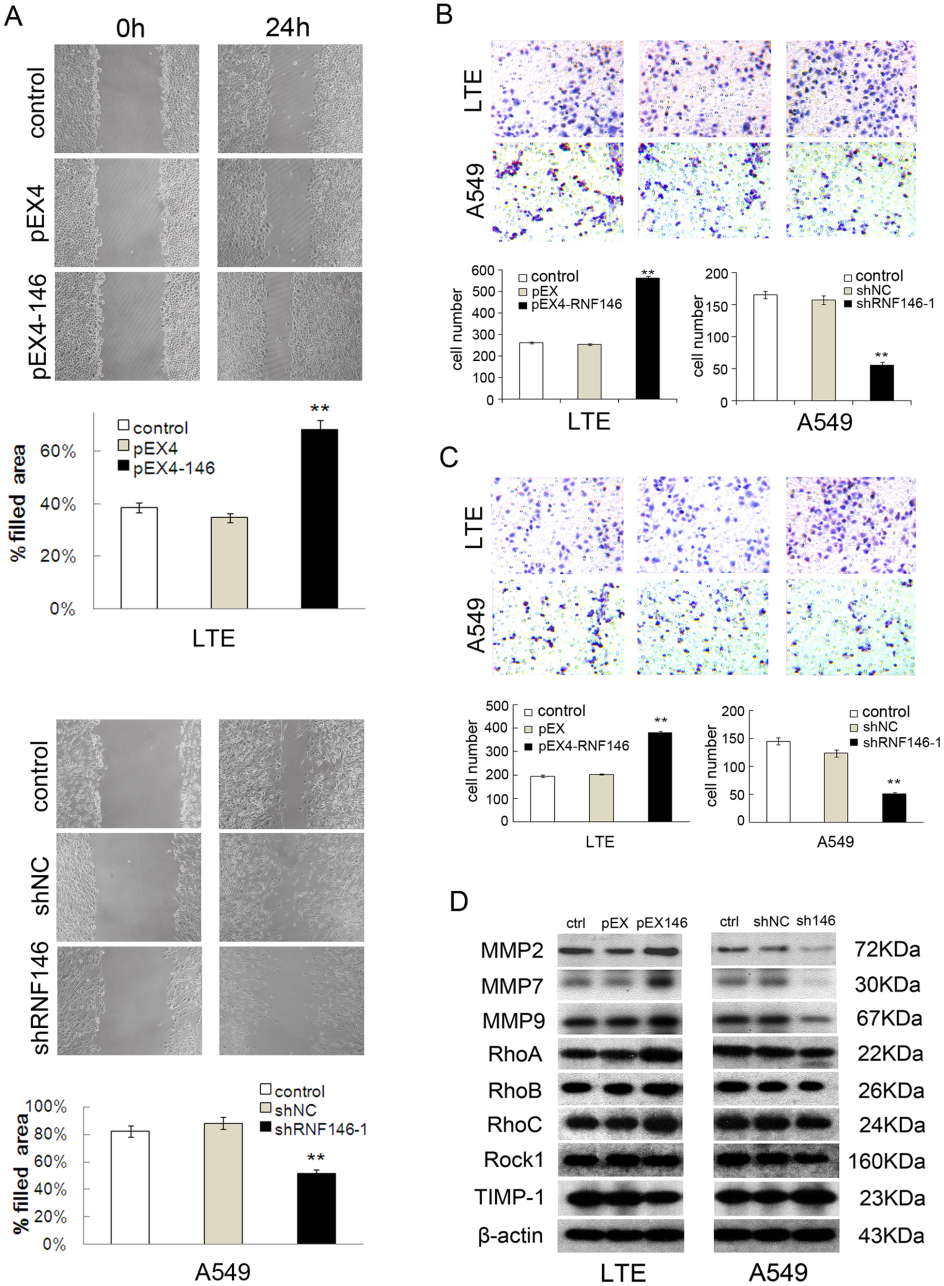
RNF146 regulated migration and invasion of lung cancer cells. (A) Wound healing assay in LTE cells overexpressing RNF146 and A549 cells treated with shRNA-1 targeting RNF146. The histogram shows the percentages of migrated cells in the scraped areas (bottom). (B–C) Analysis of cell migration and invasion ability in the transwell assay. The histogram demonstrates the number of migrated or invaded cells. (D) Western blot of migration and invasion–associated proteins. β-actin served as the internal control.

Overexpression and knockdown of RNF146 affected the cell migration and invasion abilities of NSCLC cells. Wound-healing and transwell experiments demonstrated that overexpression of RNF146 enhanced cell migration and invasion in LTE cells, and that knockdown of RNF146 decreased cell migration and invasion in A549 cells (Figure 4A, B, C). Similar results were obtained in H1299 cells in which RNF146 expression was depleted (Figure S2, S3). Proteins related to migration and invasion were also detected by Western blot. The expression levels of MMP2 and MMP7 were increased in LTE cells that overexpressed RNF146, whereas the levels of RhoA, RhoB, RhoC, Rock1, and TIMP-1 were unchanged. Furthermore, levels of MMP2 and MMP7 were decreased significantly when RNF146 expression was knocked down (Figure 4D).

### RNF146-regulated Expression of cyclinD1 and MMP7

**Figure 5:**
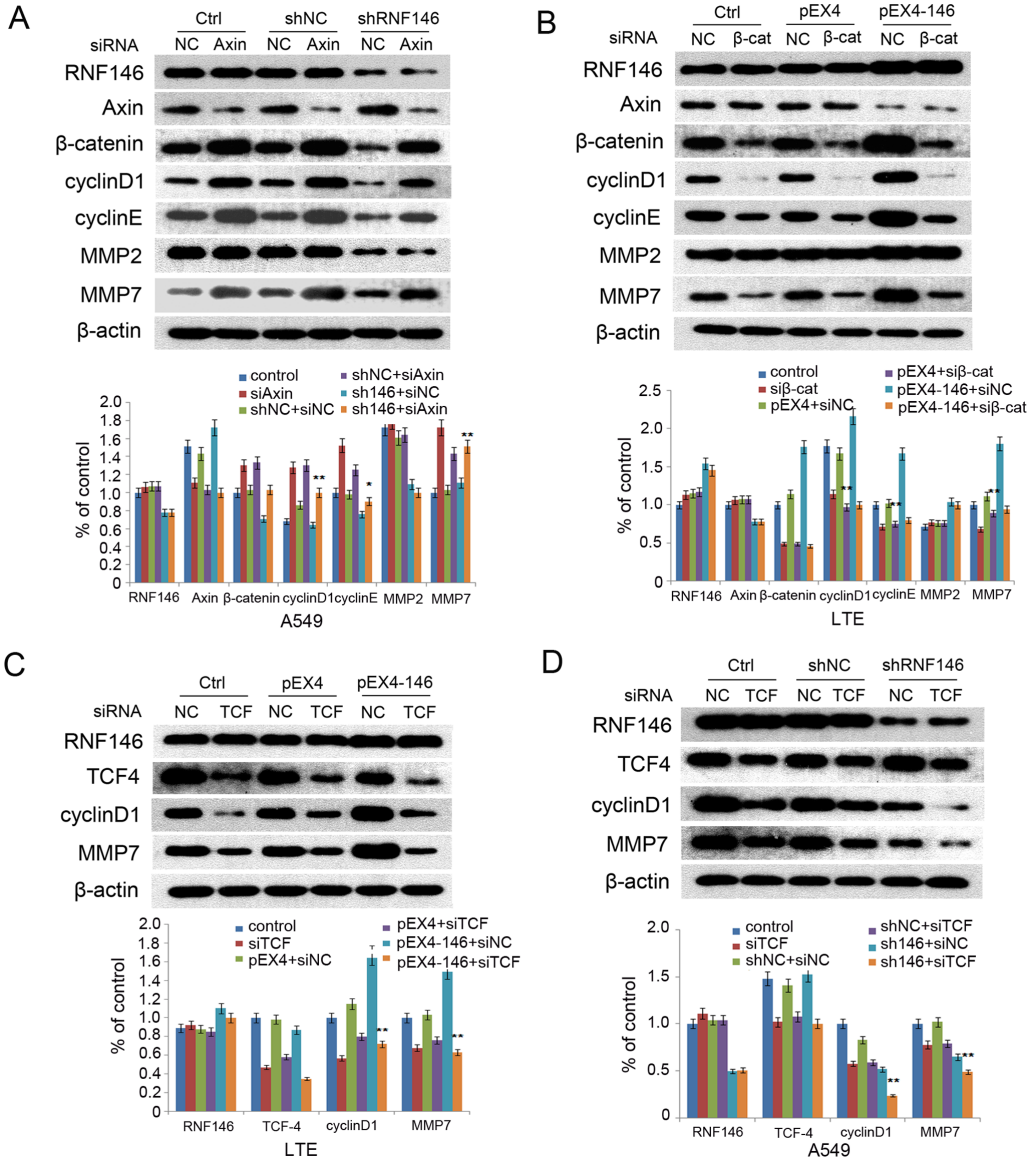
RNF146-mediated regulation of cyclinD1 and MMP7. (A) Levels of cyclinD1/E and MMP2/7 in A549 cells after treatment with shRNA targeted to RNF146 and siRNA targeted to Axin. (B) Levels of cyclinD1/E and MMP2/7 in LTE after overexpression of RNF146 and knockdown of β-catenin. (C and D) Levels of cyclinD1 and MMP7 were detected after knockdown of endogenous TCF-4 in LTE and A549 cells with overexpression or knockdown of RNF146.

We proposed that RNF146 regulated cell migration and invasion by indirectly regulating target-gene transcription through Axin/β-catenin signaling. To test this hypothesis, we knocked down RNF146 in cells treated with siRNA targeting Axin. We found that the levels of cyclinD1, cyclinE, and MMP7 recovered as compared to cells treated with shRNF146 alone (Figure 5A). Overexpression of RNF146 in combination with siRNA knockdown of β-catenin did not cause levels of cyclinD1, cyclinE, or MMP7 to increase, whereas the MMP2 levels were still increased (Figure 5B). Knockdown of endogenous TCF-4 in LTE cells combined with overexpression RNF146 did not cause levels of cyclinD1 and MMP7 to increase (Figure 5C). However, when RNF146 and TCF-4 were knocked down in A549 cells simultaneously, expression of cyclinD1 and MMP7 decreased more dramatically (Figure 5D). Taken together, the data indicated that RNF146 probably regulated expression of cyclinD1 and MMP7 by the Wnt/β-catenin signaling pathway.

## Discussion

In the present study, we found that RNF146 was overexpressed in NSCLC tissue compared to adjacent normal lung tissues. The RNF146 expression level showed correlations with several clinical pathological factors, including tumor size, differentiation level, lymphatic metastasis, pTNM staging, and the prognosis of patients in stage I, which are important factors that represent the potential for lung cancer malignancy. These data suggest that overexpression of RNF146 in NSCLC may enhance metastasis in lung cancer, and that RNF146 might be an indicator of poor prognosis in stage I NSCLC.

Our data are consistent with the findings of two recent reports [10]–[11] that RNF146 might be involved in the development of tumors. Gold *et al.* discovered a breast cancer risk gene in the Jewish Ashkenazi population located on 6q 22.33 in a genome-wide association study. This location contained two genes, *RNF146* and enoyl CoA hydratase domain containing 1 [11]. Another important discovery was that RNF146 interacted with PARsylated Axin through its PAR-binding motif, leading to Axin degradation and positive regulation of the Wnt signaling pathway. These results provided new evidence for the potential role of RNF146 in tumor development [1], [6]. It has been indicated that the newly identified RNF146 substrates basic leucine zipper nuclear factor 1 (BLZF1) and cancer susceptibility candidate 3 (CASC3) are involved in tumor development and cell proliferation [12]–[13]. These reports suggest that RNF146 might play important roles in tumors by altering gene expression or degrading target proteins.

The Wnt signaling pathway affects gene expression and cell migration. As an important molecule in the Wnt signaling pathway, Axin participates in formation of the Axin/glycogen synthase kinase 3β (GSK-3β)/adenomatous polyposis coli (APC)/Casein kinase I (CKI) degradation complex, which enhances the degradation of β-catenin and inhibits tumor proliferation, invasion, and metastasis [14]–[15]. Previous studies have established that Axin plays important roles in NSCLC [14]. Axin expression has been shown to inhibit the proliferation and invasion of lung cancer cells [9]. Cytoplasmic stabilization and accumulation of nuclear β-catenin, an important signal transducer, are important processes in Wnt signal transduction.

We found that RNF146 was negatively correlated with the expression of Axin and positively correlated with nuclear expression of β-catenin. Immunofluorescent staining demonstrated that RNF146 regulated the distribution and nuclear accumulation of β-catenin. Previous studies have demonstrated that nuclear β-catenin expression leads to the loss of cell adhesion. Moreover, this protein increased the transcription of target genes, including c-myc, cyclinD1, VEGF, and MMP7, by interacting with the T cell factor/lymphoid enhancer factor, thereby leading to the proliferation and metastasis of tumor cells [16]–[17]. In the present study, RNF146 regulated Axin and β-catenin of the Wnt signaling pathway, indicating that RNF146 affected the proliferation and invasion of tumor cells.

Our MTT and cell cycle experiments showed that RNF146 not only regulated cell proliferation and cell cycle progression but also affected the expression levels of proteins related to the cell cycle. Cyclin D1 mainly regulates G_1_ phase progression [18], and cyclin E enhances G_1_/S transition [19]–[20]. Cyclin D1 could interact with CDK4/6 and enhance the transcription of downstream genes [18]. Thus, it can be concluded that RNF146 controls tumor proliferation by regulating the cell cycle.

Our data also showed that RNF146 affected the migration and invasion of lung cancer cells, suggesting that RNF146 has multiple functions. The scratch test and transwell assay results showed that RNF146 enhanced the migration and invasion of lung cancer cells. MMPs degrade various proteins in the extracellular matrix, disrupt tissue barriers to tumor cell invasion, and play key roles in the invasion and metastasis of tumors [21]. The data in the present study revealed that MMP2 and MMP7 were regulated by RNF146. Degradation of the extracellular matrix and bone matrix by MMP2 and MMP7 has been shown to be closely related to NSCLC invasion and metastasis [22]–[23]. The data suggest that RNF146 might regulate the migration and invasion of lung cancers by regulating MMP2 and MMP7.

Prior to our study, the roles of RNF146 in the Wnt signaling pathway were confined to the degradation of Axin and the promotion of the nuclear aggregation of β-catenin. Because β-catenin interacts with TCF-4 after it enters the nucleus [16]–[17], we hypothesized that RNF146 could regulate cell proliferation and invasion by indirectly regulating targeted gene transcription through Axin/β-catenin. Our experiments showed that RNF146 did not play regulatory roles in the downstream proteins cyclinD1, cyclinE, and MMP7 in cells with knockeddown Axin and β-catenin. Similarly, overexpression or knockdown of RNF146 did not induce changes in the targeted protein levels when TCF4 was knocked down. Interestingly, the level of MMP2 was not regulated by Axin and β-catenin, indicating that MMP2 may not be a direct target of the Wnt signaling pathway, and RNF146 may regulate MMP2 by other mechanisms. The data demonstrated that RNF146 probably regulated cyclinD1 and MMP7 by the Wnt/β-catenin signaling pathway.

However some caveats need to be considered in the potential mechanism of RNF146 in Wnt signaling pathway. First, Wnt is a very complex process and lots of proteins are involved in Wnt regulation. Wnt signaling is regulated by altered expression of various regulatory proteins in lung cancer and RNF146 may be the only one of those regulators. So, further studies are required to verify the potential mechanism of RNF146 in Wnt signaling pathway. Second, RNF146 was confirmed to be the novel E3 ubiquitin ligase 1, however whether the biologic function of RNF146 in tumors is dependent or independent on ubiquitin ligase activity remains unknown. This potential link needs to be fully investigated.

In summary, we observed high expression levels of RNF146 in clinical NSCLC samples, consistent with the finding that RNF146 overexpression enhanced the proliferation and invasion of lung cancer cells. In addition to regulating the growth and survival of tumor cells, RNF146 played tumor-enhancing roles and affected the migration and invasion of tumor cells in NSCLC. These effects probably depended on key factors, including Axin and β-catenin in the Wnt signaling pathway. The results reveal new roles for RNF146 in the development and progression of NSCLC and identify RNF146 as a potential target for lung cancer treatment.

## Supporting Information

Figure S1 **Silencing of RNF146 suppressed cell growth and regulated cell cycle progression.** (A) Silencing of RNF146 in A549 and H1299 cells. Both cell lines were transfected with shNC, shRNF146-1, or shRNF146-2. After 48 hours, the effective knockdown of RNF146 was confirmed by Western bolt. (B) MTT assay of A549 and H1299 cells showed decreased number of viable cells by shRNA-mediated knockdown of RNF146. **P<0.05. (C) Effects of RNF146 depletion on cell cycle analyzed by flow cytometry.
(TIF)

Figure S2 **Knockdown of RNF146 inhibited cell migration.** Wound-healing assay was carried out. Confluent monolayer of A549 cells (A) or H1299 cells (B) was scratched using a sterile pipette. At 24 hours after wounding, migration of cells into the scraped area was photographed. The percentages of migrated cells were quantified. **P<0.05.
(TIF)

Figure S3 **Effects of RNF146 on migration and invasion in A549 and H1299 cells.** Cell migration and invasion assays of A549 and H1299 cells transfected with shNC, RNF146-specific shRNA were carried out using a 12-well Transwell. The images show representative density of cells that migrated (A, C) or invasion (B, D). The bar graph depicts quantification of migration or invasion cells. **P<0.05.
(TIF)
