# Supplementary figures and images for: Overexpression of RNF146 in Non-Small Cell Lung Cancer Enhances Proliferation and Invasion of Tumors through the Wnt/β-catenin Signaling Pathway
Source: PLoS One. 2014 Jan 14;9(1):e85377. doi: 10.1371/journal.pone.0085377 (PMC3891871; doi:10.1371/journal.pone.0085377)

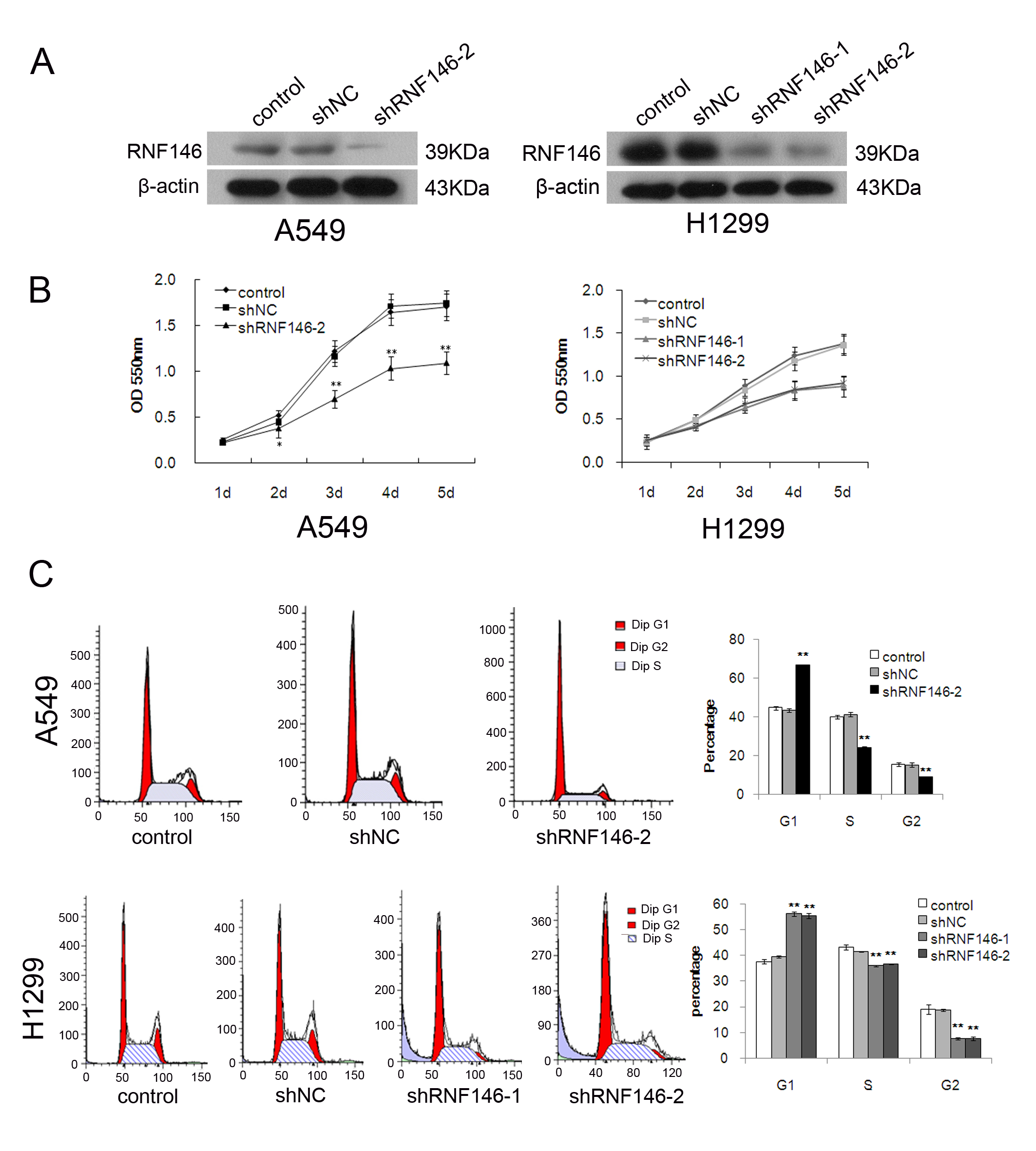

Supplement: Figure S1 — Silencing of RNF146 suppressed cell growth and regulated cell cycle progression. (A) Silencing of RNF146 in A549 and H1299 cells. Both cell lines were transfected with shNC, shRNF146-1, or shRNF146-2. After 48 hours, the effective knockdown of RNF146 was confirmed by Western bolt. (B) MTT assay of A549 and H1299 cells showed decreased number of viable cells by shRNA-mediated knockdown of RNF146. **P<0.05. (C) Effects of RNF146 depletion on cell cycle analyzed by flow cytometry. (TIF) [file pone.0085377.s001.tif]

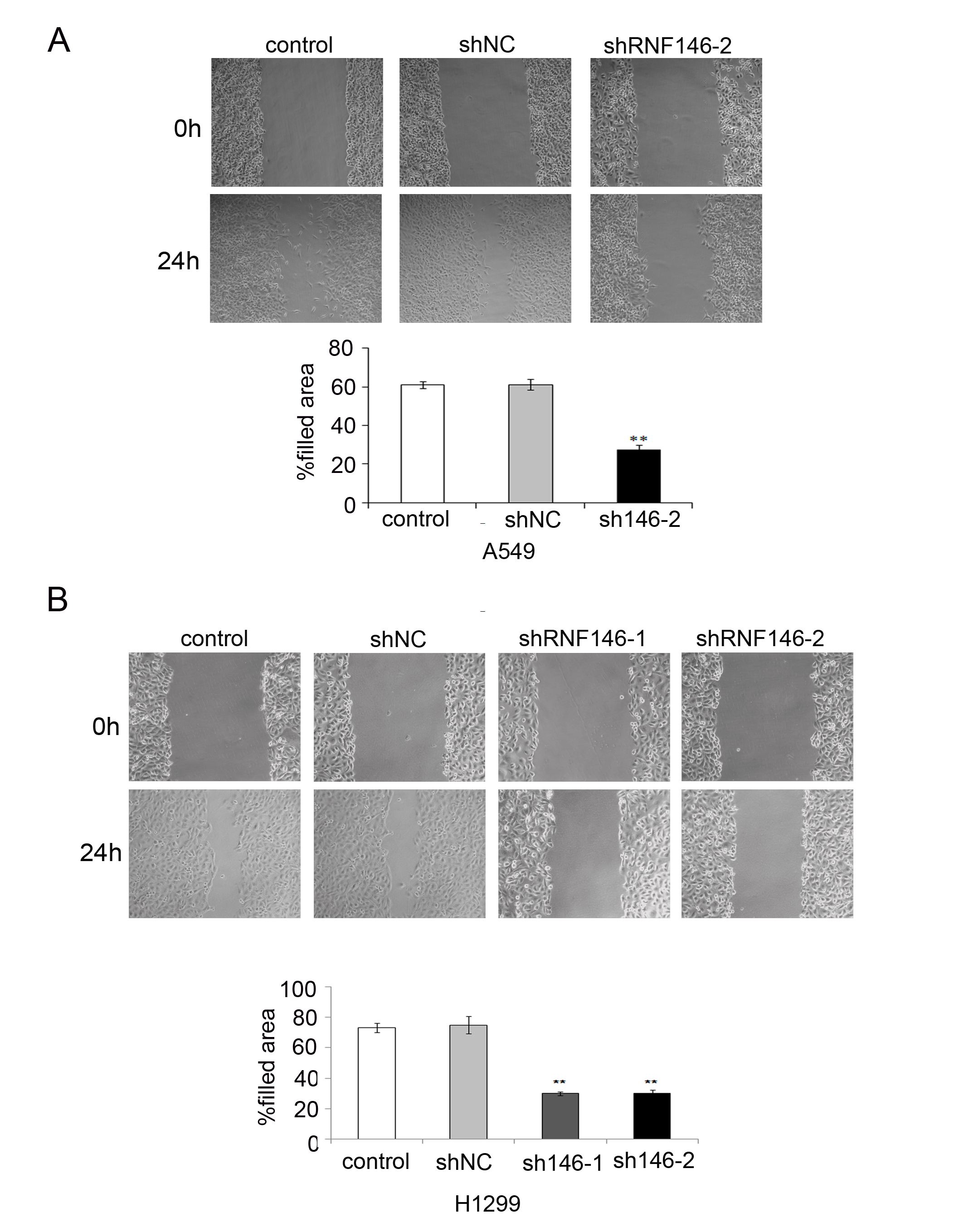

Supplement: Figure S2 — Knockdown of RNF146 inhibited cell migration. Wound-healing assay was carried out. Confluent monolayer of A549 cells (A) or H1299 cells (B) was scratched using a sterile pipette. At 24 hours after wounding, migration of cells into the scraped area was photographed. The percentages of migrated cells were quantified. **P<0.05. (TIF) [file pone.0085377.s002.tif]

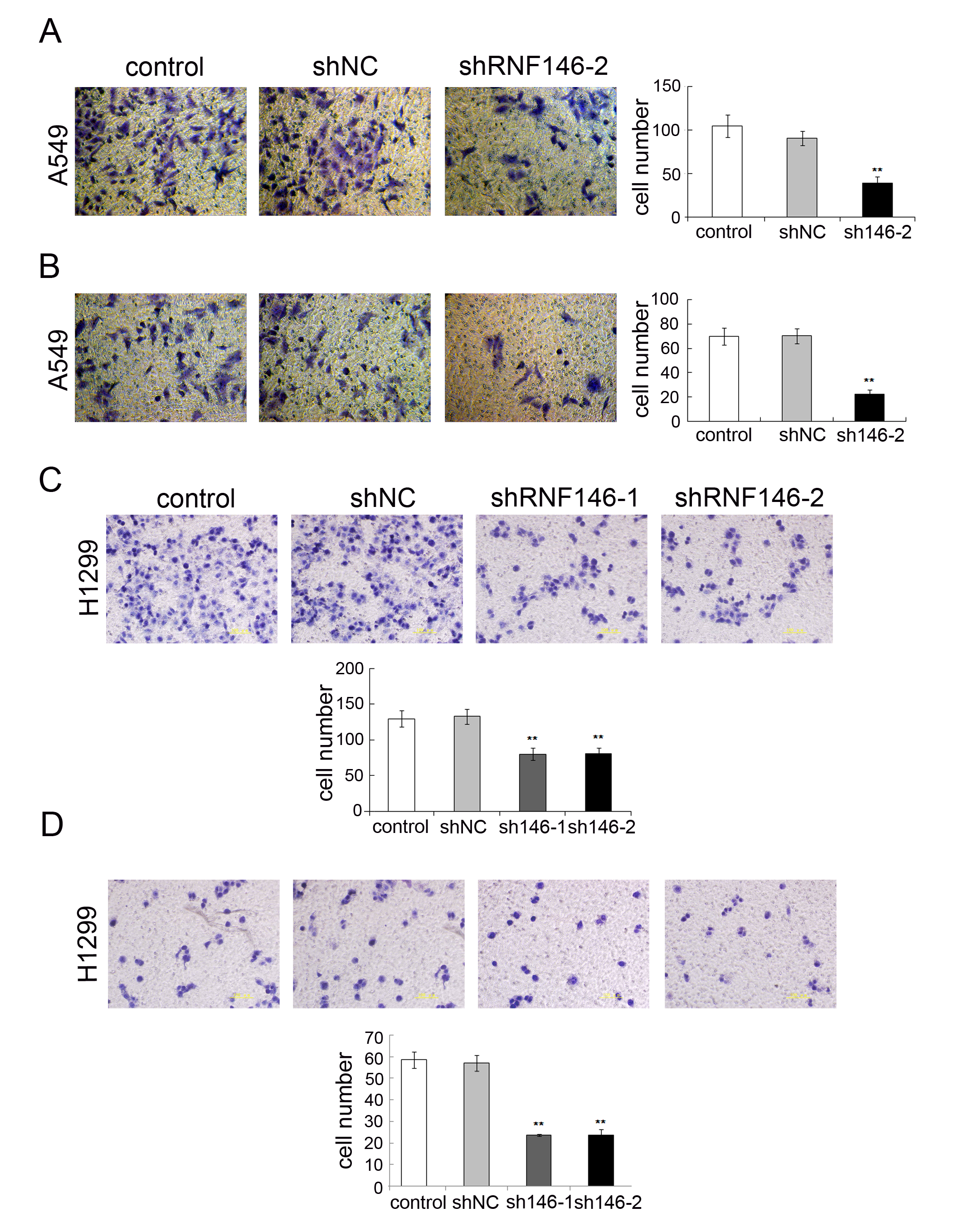

Supplement: Figure S3 — Effects of RNF146 on migration and invasion in A549 and H1299 cells. Cell migration and invasion assays of A549 and H1299 cells transfected with shNC, RNF146-specific shRNA were carried out using a 12-well Transwell. The images show representative density of cells that migrated (A, C) or invasion (B, D). The bar graph depicts quantification of migration or invasion cells. **P<0.05. (TIF) [file pone.0085377.s003.tif]
